# Action Potential-Evoked Calcium Release Is Impaired in Single Skeletal Muscle Fibers from Heart Failure Patients

**DOI:** 10.1371/journal.pone.0109309

**Published:** 2014-10-13

**Authors:** Marino DiFranco, Marbella Quiñonez, Perry Shieh, Gregg C. Fonarow, Daniel Cruz, Mario C. Deng, Julio L. Vergara, Holly R. Middlekauff

**Affiliations:** 1 Department of Physiology, David Geffen School of Medicine, University of California Los Angeles, Los Angeles, California, United States of America; 2 Department of Neurology, David Geffen School of Medicine, University of California Los Angeles, Los Angeles, California, United States of America; 3 Division of Cardiology, Department of Medicine, David Geffen School of Medicine, University of California Los Angeles, Los Angeles, California, United States of America; University of Canberra, Australia

## Abstract

**Background:**

Exercise intolerance in chronic heart failure (HF) has been attributed to abnormalities of the skeletal muscles. Muscle function depends on intact excitation-contraction coupling (ECC), but ECC studies in HF models have been inconclusive, due to deficiencies in the animal models and tools used to measure calcium (Ca^2+^) release, mandating investigations in skeletal muscle from HF patients. The purpose of this study was to test the hypothesis that Ca^2+^ release is significantly impaired in the skeletal muscle of HF patients in whom exercise capacity is severely diminished compared to age-matched healthy volunteers.

**Methods and Findings:**

Using state-of-the-art electrophysiological and optical techniques in single muscle fibers from biopsies of the locomotive *vastus lateralis* muscle, we measured the action potential (AP)-evoked Ca^2+^ release in 4 HF patients and 4 age-matched healthy controls. The mean peak Ca^2+^ release flux in fibers obtained from HF patients (10±1.2 µM/ms) was markedly (2.6-fold) and significantly (p<0.05) smaller than in fibers from healthy volunteers (28±3.3 µM/ms). This impairment in AP-evoked Ca^2+^ release was ubiquitous and was not explained by differences in the excitability mechanisms since single APs were indistinguishable between HF patients and healthy volunteers.

**Conclusions:**

These findings prove the feasibility of performing electrophysiological experiments in single fibers from human skeletal muscle, and offer a new approach for investigations of myopathies due to HF and other diseases. Importantly, we have demonstrated that one step in the ECC process, AP-evoked Ca^2+^ release, is impaired in single muscle fibers in HF patients.

## Introduction

Chronic heart failure (HF) patients with systolic dysfunction have benefited from advances in pharmacological and device therapies that have reduced mortality [1]. Nonetheless, residual poor exercise tolerance characterized by skeletal muscle weakness and early fatigue contribute to ongoing suffering and morbidity, leading to 3.5 million hospitalizations per year, 11 million out-patient doctor visits/year, and skyrocketing medical costs [2]. It is a fact that a large proportion of HF patients would trade increased length of life for increased quality of life [3], which in HF patients is directly linked to exercise capacity [4]. Surprisingly, exercise dysfunction in HF patients is not directly related to severity of left ventricular dysfunction, intra-cardiac hemodynamics at rest or during exercise, or inadequate skeletal muscle blood flow [5]–[8]. Instead, exercise dysfunction is directly related to abnormalities of the skeletal muscles themselves; in fact a skeletal myopathy has been described which includes a fiber shift from type I aerobic fibers to type II anaerobic fibers and decreased mitochondrial volume and metabolic function [8]–[11].

Skeletal muscle function depends critically on both intact energy metabolism and on the robustness of the sequence of events linking the electrical with the mechanical activation of the muscle fibers (the so-called excitation-contraction coupling [ECC] process). The role of metabolic deficiencies in skeletal muscle function in HF patients on optimal medical therapy has recently been questioned [12]–[14]. We have recently reported marked abnormalities of several key proteins participating in the ECC process in humans with HF [15]; however, it remains unknown whether these abnormalities in protein content are accompanied by functional abnormalities in calcium (Ca^2+^) cycling in humans with HF. The integrity of important steps in the ECC chain of events, such as the Ca^2+^ release from the sarcoplasmic reticulum (SR), is a decisive factor for normal exercise ability; ECC impairments are expected to critically contribute to muscle weakness and poor exercise tolerance [8], [14]–[16].

Animal models of HF have been used to investigate ECC abnormalities that may recapitulate the skeletal myopathy seen in HF patients [17]–[22]. However, these animal studies have intrinsic limitations in that they provide inconsistent information depending on the HF model and they do not fully reproduce the HF phenotype of decreased exercise tolerance [23]. Furthermore, to date, tools capable of measuring the rapid physiological Ca^2+^ release in skeletal muscle fibers have not been utilized in animal models. Altogether, the findings from animal studies are inconclusive and contradictory [18]–[22]. We chose to bypass the animal models and procure biopsies of the *vastus lateralis* (VL) muscles directly from HF patients and healthy age-matched volunteers, and used state-of-the-art electrophysiological and optical techniques to test our hypothesis that the ECC process is significantly impaired in the locomotive skeletal muscle of HF patients compared to healthy volunteers. Our approach puts special emphasis on the fibers’ ability to generate Ca^2+^ transients in response to action potential (AP) stimulation and, by using low affinity Ca^2+^ indicators in conjunction with high internal EGTA concentrations [17]–[20], allows for a direct comparison of their physiological robustness.

## Methods

### Ethics Statement

The study was approved by the University of California Los Angeles (UCLA) Human Subjects Protection Committee, and the participants gave their written informed consent.

### Study Population

Advanced HF patients, New York Heart Association Class II–III meeting the following eligibility criteria were recruited from the Ahmanson-UCLA Cardiomyopathy Center: 1) age 21–65 years, 2) Left ventricular ejection fraction <35%, 3) HF duration > 1 year, 4) not involved in a formal exercise training program, 5) on stable, guideline appropriate HF medications, and not taking warfarin or non-warfarin oral anticoagulants. Age and gender-matched healthy, non-smoking controls, without chronic illnesses and taking no daily medications, who drank <2 drinks/day, and who did not participate in regular exercise (30 minutes most days), served as controls.

### Skeletal muscle biopsy

The procedure was performed at the UCLA Out-Patient Surgery Center under local anesthesia. An incision was made in the lateral aspect of the thigh, at about 20 cm proximal to the patella, and a sample of vastus lateralis (VL) muscle measuring 40 mm in length, 8 mm in width and 5 mm in depth was obtained. The biopsy was transported to the lab in ice-cold oxygenated Tyrode solution, where it was pinned in a Sylgard bottomed Petri dish containing oxygenated Tyrode solution and divided into 10–12 bundles (∼2 mm in diameter), as delimited by the perimysium. Bundle pulling was kept at a minimum at all times during dissection. When kept in oxygenated Tyrode solution at room temperature, bundles remained viable for >10 hours. Although not studied systematically, viability was found to be longer the greater the biopsy length. In many cases, bundles 40 mm in length contracted upon stimulation with 10 Hz trains after 1.5 hours from biopsy.

### Muscle fiber dissection

A bundle was pinned down at slack length in a dish containing Tyrode, which was subsequently slowly exchanged by relaxing solution (see Solutions). In this solution, fibers became electrically and mechanically inactivated, thus preventing contractures during dissection and increasing the rate of success of intact fiber isolation. A peripheral smaller bundle comprised of ∼20 fibers was dissected aside using small Vannas scissors and #5 tweezers. The tissue was divided progressively into smaller bundles until a single fiber of about 20 mm in length was obtained. For this finer dissection, #5 tweezers and retina surgical scissors (gauge 25, Alcon) were used. Fiber stretching was avoided at all times. Only fiber segments crystalline in appearance, devoid of contractured regions, freely flexible, and exhibiting a sharp banding pattern were used for electrophysiological experiments. The degree of difficulty of dissection of fibers from HF biopsies was not significantly different from that of control biopsies.

### Fiber mounting and stimulation

Fibers were mounted in an inverted double-gap grease chamber, under stereoscopic microscope observation, as previously described [24], [25] but with modifications. Fibers (∼8 mm in length) were transferred to the chamber previously flooded with relaxing solution using a heat polished glass capillary connected to a 100 µl pippetor via a Tygon tubing. To avoid adhesion of the fiber to the capillary, it was rinsed in relaxing solution containing 3 mg/ml serum bovine albumin prior to fiber transfer. Using #5 tweezers the fiber was placed across the chamber’s gaps, where pre-seals (a shallow layer of grease) had been constructed. Each end of the fiber was secured to the coverslip at the chamber’s lateral compartments with grease, and straightened and stretched by ∼ 40–50% the slack length by gently pulling the ends embedded in grease. The grease seals were then constructed at the chamber’s gaps (Figure 1 in [24]), and the excess solution was removed to render each of the 3 compartments electrically independent. The central pool of the experimental chamber was 300±45 µm (n = 25). The segments of the fiber at the lateral compartments were permeabilized with saponine (10 µg/ml in internal solution, 30 sec). After washing out saponine, internal solution containing the Ca^2+^ sensor was added to the lateral compartments. Finally, the chamber was placed on the stage of the microscope and connected to the perfusion system and the electrical amplifier. Fibers requiring more than −70 nA to be repolarized to −90 mV were discarded. Recordings were started 15–20 min after adding the Ca^2+^ sensor to the lateral pools. The circuit used to stimulate the fibers and to record the membrane potential and currents has been previously described [25]. One cut end of the fiber was connected to a current source, the other was connected to a high impedance amplifier, and the central segment of the fiber was held at virtual ground. APs were elicited by current pulses (0.2 ms, 10–15% above threshold) applied at one cut end. Membrane potential was measured as the difference between the potential at the other cut end (i.e. the myoplasm) and the central pool (i.e. the extracellular milieu).

**Figure 1:**
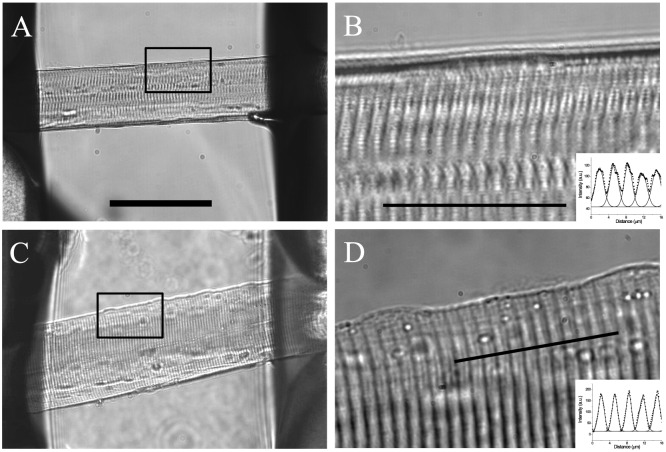
Isolated fibers mounted in the inverted double grease-gap chamber. Panel A: Image of a VL fiber from a healthy volunteer obtained with a 20× objective. The inner part of the grease seals delimiting the experimental pool can be seen at both sides of the image. Panel B: The area delimited by the square in A was imaged with a 100× immersion objective. A sharp banding pattern and smooth border can be seen. Panels C and D shows a VL fiber from a HF patient at the same magnifications as in A and B, respectively. The diameter and sarcomere length were 62.0 and 3.0 µm for the fiber in A, and 80 and 2.8 µm for the fiber in B, respectively. The insets in panels B and D show intensity profiles measured across ∼4 sarcomeres. The profiles span ∼16 mm along the directions indicated by the lines superimposed in the corresponding images. The dots and lines represent the intensity values and Gaussian fits to the data, respectively. The scale in A corresponds to 100 µm in A and C, and 20 µm in B and D.

### Ca^2+^ transients

AP evoked Ca^2+^ concentration changes were measured using the low affinity dye OregonGreenBapta488-5N (OGB-5N) [26]–[29]. OGB5N was added to the internal solution bathing both cut ends of the fibers at a final concentration of 250µM. Large concentrations of EGTA semi-saturated with Ca^2+^ were added to the internal solution with two purposes in mind: a) to prevent fiber movements that would distort optical recordings and potentially dislodge the grease seals; and, b) to allow for the calculation of peak Ca^2+^ flux from OGB-5N Ca^2+^ transients using a single compartment model that is extensively described elsewhere [27], [28], [30]. Two mixtures were used: 30∶15 and 20∶10 EGTA:Ca. The large [EGTA] used are expected to equalize the resting myoplasmic free [Ca] to ∼80 nM in all experiments. The Ca^2+^ dependent fluorescence transients (referred to simply as Ca^2+^ transients) are reported as ΔF/F transients (dimensionless). These transients are used to calculate Ca^2+^ flux transients (μM/ms).

The optical setup was similar to that described elsewhere [28], [29]. Briefly, it consisted of an inverted microscope (Olympus IX71) equipped with a standard epifluorescence attachment, a 480-40/510/540-30 nm cube combination, and a 100× 1.4 NA oil immersion objective. The light was focused to a spot with a diameter similar to that of fibers (40–50 µm). In order to reduce stray fluorescence from the grease, the illumination spot was place at the center of the experimental pool. To remove contributions from stray fluorescence to the OGB5N fluorescence, the fluorescence from areas outside the fiber was recorded and subtracted from every Ca^2+^ transient before calculating ΔF/F transients.

### Solutions

The composition of solutions (mM) was:

Tyrode: 150 NaCl, 4KCl, 1MgCl_2_, 2CaCl_2_, 10 glucose, 10 MOPS.

Relaxing solution: 120 K_2_SO_4_, 10 MOPS, 1 MgCl_2_, 1 CaCl_2_, 10 glucose.

Internal solution: 92 K-Aspartate, 20 MOPS, 1 MgCl_2_, 5 reduced Glutathione, 5 ATP-TRIS, 5 Creatine Phosphate-Na_2_, 20 EGTA, 10 Ca(OH)_2_.

All solutions were adjusted to ph = 7.4 and had 300±5 mOsmol/kg H_2_O.

### Signal conditioning and statistical analysis

Voltage, current and optical signals were filtered at 10, 5 and 2 kHz (8 pole low-pass-filter; Frequency Devices), respectively, and acquired at 10µs/point. Optical and electrical data from control and HF fibers were compared using the Student’s t test. Significance was set at p<0.05.

## Results

### Study population characteristics

Four HF patients at an advanced stage, and four healthy age-matched human volunteers participated in these studies. The major features of the cohorts are presented in Table 1. Importantly, HF patients and healthy volunteers did not differ in age, sex, or body mass index. In addition, HF patients had severe exercise limitation as measured by peak oxygen consumption of 13.1±1.0 ml/kg/min (compared with that of untrained healthy adult values, always >28 ml/kg/min).

**Table 1:** Study Population Characteristics.

	Subjects	
	Heart Failure Patients	Healthy Volunteers
Number of Subjects	4	4
Age (years)	57.3±2.1	52.8±2.4
BMI^a^ (kg/m^2^)	29.2±1.6	30.8±2.9
Female	1	1
Druation of HF (years)	8.5±2.2	
Peak VO_2_ (ml/kg/min)	13.1±1.0	
LVEF^c^(%)	26.0±4.0	
Diabetes mellitus	2	
Hypertension	2	
Etiology of HF		
• CAD^d^	2	
• Idiopathic	2	
• Familial	0	
Medications		
• Beta-blockers	4	
• ACEI^e^	2	
• ARB^f^	1	
• Statin	4	
• Aldosterone antagonist	2	
• Aspirin	3	
• Clopidogrel	0	
• Furosemide	3	
• Digoxin	1	
• Amiodarone	1	

a body mass index, ^b^peak oxygen consumption, ^c^left ventricular ejection fraction, ^d^coronary artery disease, ^e^angiotensin inhibitor, ^f^angiotensin receptor blocker. Values are mean±SEM.

### Fibers dissected from VL muscle biopsies of healthy volunteers and HF patients appear similarly intact under the light microscope

In contrast to previous reports in rat muscles [19], we found that dissecting single fibers from VL muscle biopsies was not more cumbersome when they were obtained from HF patients compared to healthy volunteers. Moreover, as illustrated in Figure 1, bright-field images demonstrate that there are no obvious differences in appearance between fibers obtained from healthy volunteers (Figures 1A and B) and HF patients (Figures 1C and D) as they are mounted in the experimental chamber. Specifically, both sets of fibers have comparable diameters. The average diameters (mean±SD) were 75.2±17.2 µm [n = 10; minimum 58.8 µm, maximum = 113.1 µm) for fibers from healthy volunteers, and 75.5±16.4 µm (n = 5; minimum 51.8 µm, maximum = 103.0 µm) for fibers from HF patients. In addition, the fibers exhibited equally sharp sarcomere banding with no indications of contractures or vacuolation suggestive of obvious structural damages. Further, the sets of fibers from HF patients and controls had comparable sarcomere lengths, 3.31±0.34 µm, (range 2.64–3.71 µm) versus 3.21±0.33 µm, (range 2.76–3.69 µm), respectively, confirming that they did not undergo gross contractures. It should be noted that the mean sarcomere length is larger than typical slack sarcomere length (∼2.2 µm) because during the mounting process, fibers are slightly stretched (see methods). Even in a more advanced inspection of the structural integrity of the transverse tubular system in live fibers stained with the fluorescence dye di-8-ANEPPS fibers [31], there were no detectable alterations in either set of fibers (see Figure S1).

### Fibers dissected from VL muscle biopsies of healthy volunteers and HF patients are comparably healthy in electrical terms

**Figure 2:**
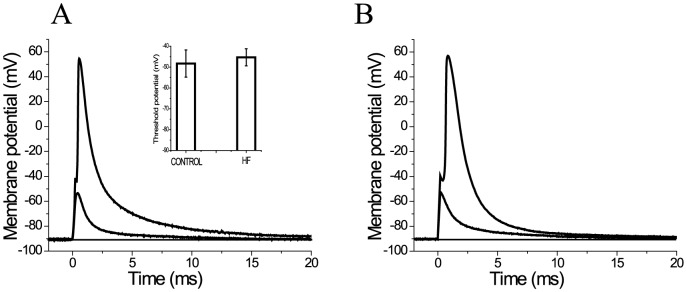
Fibers from healthy volunteers and HF patients have similar thresholds. Panels A and B show electrotonic responses (small depolarizations elicited by sub-threshold current pulses) and actions potentials (large autoregenerative depolarization elicited by suprathreshold current pulses) in a fiber from a healthy volunteer (A) and a HF patient (B). The inset in panel A shows the mean (±SD) threshold potentials for a population of fibers from healthy volunteers (n = 10 fibers) and HF patients (n = 10 fibers).

Additional direct evidence for the fibers’ intactness was obtained by measuring their spontaneous repolarization after exchanging the high K relaxing solution with Tyrode in the central compartment of the experimental chamber. Isolated fibers from both healthy volunteers and HF patients repolarized spontaneously to a similar membrane potential (−13.6±3.42 mV [n = 8] and −16.5±5.1 mV [n = 10] respectively), and required almost identical holding currents in order to maintain them at a resting potential of −90 mV (−59.2±18.3 nA [n = 8] and −60.8±28.5 nA [n = 10] respectively), and V_r_ changed only slightly throughout the typical 30 min of electrophysiological experimentation. Interestingly, these values are comparable to those previously measured in frog fibers mounted in a similar experimental chamber [24], [25], which are known to be resilient to mechanical dissection. We also determined that all fibers responded to pulses of increasing amplitude with a sharp threshold, separating electrotonic from all-or-none responses (i.e. APs). Examples of such responses obtained from a healthy volunteer and a HF patient are shown in Figures 2A and 2B, respectively. When relatively small current pulses (sub-threshold) are applied, similar graded electrotonic responses are elicited in both preparations, which peak at about −50 mV. By increasing the pulse amplitude by 10–15%, comparable all-or-none responses are evoked, which take off from about −40 mV. Threshold potentials were found between these two values. Although the threshold for HF fibers is slightly more depolarized (∼3 mV) than healthy fibers, the mean values (±SD) calculated from 10 healthy fibers (−48.3±6.5 mV) and 10 HF fibers (−45.3±4.1 mV) were not significantly different (inset of Figure 2A).

### AP-evoked Ca^2+^ release is impaired in isolated fibers from HF patients

**Figure 3:**
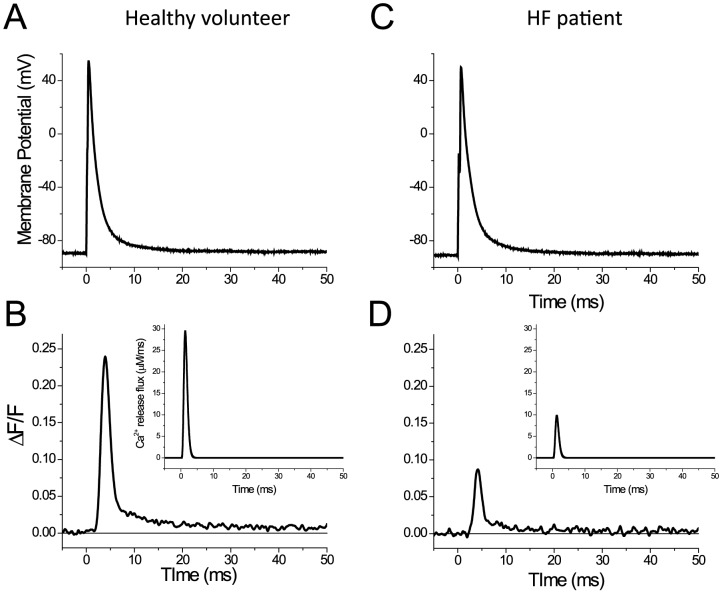
AP evoked Ca^2+^ transients in fibers dissected from a healthy volunteer and a HF patient. Figures A and C are the APs and panels B and D are the corresponding Ca^2+^ transients. The insets in B and D are Ca^2+^ release fluxes (in μM/ms) calculated from the corresponding optical records. The fibers were equilibrated with an internal solution containing 30∶15 EGTA:Ca^2+^.

Secure that fibers dissected from biopsies of both HF patients and healthy volunteers were equally intact for study in isolation, we proceeded to investigate their respective ability to release Ca^2+^ in response to AP stimulation. A smaller population of fibers was used for this purpose (5 fibers from healthy volunteers and 9 fibers from HF patients). The fibers were allowed to equilibrate with internal solutions containing high [EGTA] and free Ca^2+^ concentration [Ca^2+^] close to the physiological value ∼0.1 µM [26], [28]. Figure 3B shows an example of a Ca^2+^ transient recorded in a fiber from a healthy volunteer and equilibrated with internal solution containing 30∶15 EGTA:Ca (see Solutions). In response to the AP (Figure 3A), a fast fluorescence transient (Ca^2+^ transient) was elicited that reached a peak ΔF/F of ∼0.24. Interestingly, knowing the concentration and Ca^2+^ binding properties of the Ca^2+^ indicator (OGB-5N) and EGTA, we were able to estimate with accuracy the Ca^2+^ release flux (in μM/ms) responsible for this fluorescence transient [26], [28]–[30], [32]. The resulting flux record is shown in the inset of Figure 3B; the peak value, representing the maximal rate of Ca^2+^ release, was ∼29 µM/ms. In comparison, Ca^2+^ transients recorded from a muscle fiber dissected from a HF patient’s VL biopsy under the same conditions as those used for the fiber from the healthy volunteer were ∼3-fold smaller amplitude (peak ΔF/F∼0.09, Figure 3D). The calculated Ca^2+^ release flux (compare insets of Figures 3B and 3D) was also markedly reduced, ∼3-fold (from ∼29 to ∼10 µM/ms).

**Figure 4:**
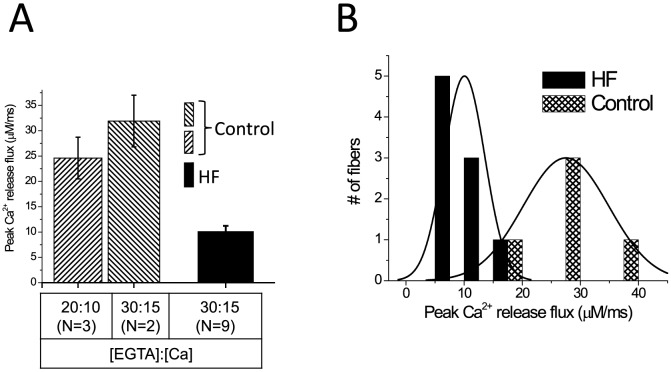
Average values and frequency distribution of peak Ca^2+^ release flux values in fibers from healthy volunteers and HF patients. Panel A shows the mean (±SD) of peak Ca^2+^ flux in fibers of healthy volunteers (hatched) and of HF patients (black) equilibrated with internal solution containing either 30∶15 or 20∶10 EGTA:Ca. Panel B shows the frequency distribution of peak Ca^2+^ release fluxes from all fibers in panel A. The binning width was 2.5 µM/ms and the lines correspond to fits of the data to standard normal distributions.

This impairment in AP-evoked Ca^2+^ transients was observed in every HF fiber studied, regardless of the EGTA:Ca ratio used to equilibrate control fibers. In order to compare reliably the Ca^2+^ release strength in all fibers studied, we calculated the Ca^2+^ fluxes underlying Ca^2+^ transients recorded at either 30∶15 or 20∶10 (EGTA:Ca) conditions. As demonstrated in Figure 4A, the mean Ca^2+^ fluxes determined in fibers from healthy volunteers equilibrated with 30∶15 (n = 2) and 20∶10 (n = 3) EGTA mixtures were comparable to each other. Since the mean flux from fibers was independent of the EGTA:Ca ratio, the data from healthy volunteers was pooled. The results demonstrate that the mean peak Ca^2+^ release flux in fibers obtained from HF patients equilibrated with a 30∶15 EGTA:Ca mixture (10±1.2 µM/ms; 4 biopsies, 9 fibers) is substantially (2.6-fold) and significantly (p<0.05) smaller than that in fibers from healthy volunteers (28±3.3 µM/ms; 4 biopsies, 5 fibers). We then compared the frequency distribution of peak Ca^2+^ fluxes from healthy volunteers and HF patients (Figure 4B). The values for AP-evoked Ca^2+^ release fluxes in fibers from healthy volunteers are widely distributed between values as high as ∼40 µM/ms to low values of ∼18 µM/ms. In contrast, those of fibers from HF patients span a much narrower, left-shifted range of lower values, between ∼17 and ∼5 µM/ms. AP-evoked Ca^2+^ release fluxes were smaller in all the fibers isolated from HF patients, compared to those from healthy volunteers. Our results demonstrate for the first time that a key step in the ECC process, that is, Ca^2+^ release from the SR, is severely impaired in skeletal muscle fibers of HF patients compared to age-matched healthy volunteers. These findings are consistent with, and expand on, our previous report that the expression of key proteins involved in ECC are reduced in HF fibers [15], to demonstrate that the deficiency in the expression of those proteins has devastating functional implications for HF patients.

### Properties of the AP in fibers isolated from biopsies of healthy volunteers and HF patients

Since the AP is the physiological trigger of the ECC process, one potential mechanism underlying the severe reduction in the Ca^2+^ release fluxes observed in fibers from HF patients could be that their AP properties are significantly altered. To our knowledge, there is no published information about the electrical activity of human VL muscle fibers; consequently, we compared the amplitude, full duration at half-maximum (FDHM), time to peak (TTP), and maximal rate of depolarization ([dV/dt]_max_) of APs recorded in fibers isolated from the skeletal muscle biopsies of HF patients and healthy volunteers (see Table 2). The APs of fibers dissected from biopsies of healthy volunteers and HF patients have almost identical amplitudes and durations. Since the resting potential was kept at −90 mV in both groups of fibers, their APs have similar overshoots (to ∼+54 mV). Interestingly, the maximum depolarization rate was significantly faster in fibers from HF patients compared to healthy volunteers (p<0.05). Potential implications of this faster depolarization will be addressed in the Discussion.

**Table 2:** Properties of action potentials recorded in fibers from healthy volunteers and HF patients.

	Subjects	
	Heart Failure Patients	Healthy Volunteers
Number of Subjects	4	4
Number of fibers	10	10
AP amplitude (mV)	143.5±3.2	144.1±3.9
FDHM^a^ (ms)	2.19±0.16	2.18±0.16
TTP^b^ (ms)	0.68±0.16	0.62±0.09
dV/dt (m/s)	791.3±83.0*	647.5±57.7*
Threshold (mV)	−45.3±4.1	−48.3±6.5

For all fibers, the resting potential was maintained at −90 mV. ^a^Full-duration-at-half-maximum, ^b^time to peak. Values given as Mean±SEM. * HF vs healthy volunteer, p<0.05.

## Discussion

Using state-of-the-art electrophysiological and optical techniques, we have performed studies in single fibers procured from the *vastus lateralis* muscle of advanced HF patients and healthy volunteers, and have demonstrated for the first time that one critical step of the ECC process, specifically, the AP-evoked Ca^2+^ release, is impaired in this locomotive muscle of HF patients. As will be discussed in detail below, these findings are transformative because: 1) they circumvent the inherent limitations of studying ECC in models of HF by utilizing actual tissue obtained directly from patients afflicted with the HF condition; 2) they confirm the feasibility of performing electrophysiological experiments characterizing individual steps of the ECC process in single fibers from human skeletal muscle according to fiber type; and critically, 3) they offer new approaches to investigate other myopathic conditions that may also involve impairments in the ECC process, such as chronic obstructive pulmonary disease and chronic renal disease [33], [34].

### Inherent limitations in animal models and the need for human studies

Animal models of HF, including the infarct model and the sympathetic hyperactivity-induced model, are critical to our further investigations and improved understanding of human diseases, but have inherent limitations [17]–[23]. In the rat infarct model, the left coronary artery is ligated, which results in infarction of ∼35% of cardiac tissue and elevated left ventricular end-diastolic pressures, mimicking human HF. However, despite significant cardiac damage and dysfunction, HF rats demonstrate no decline in locomotive activity when quantified by the photocell activation technique [23]. That is, the phenotypic expression of decreased exercise tolerance and early fatigue is absent in this model, thereby raising the question of its validity to study exercise dysfunction in patients with HF who suffer from profound exercise limitation. The sympathetic hyperactivity-induced HF model is a congenic α_2A_/α_2C_ adrenoceptor knockout mouse that leads to sympathetic hyperactivity-induced HF and quantified exercise dysfunction [18]. However, since α_2A_/α_2C_ adrenoceptors are absent in all tissues, including the skeletal muscle, it is difficult to know whether muscle changes are due to HF, or to the genetic modification. In fact, in this model, the sympathetic hyperactivity leads to early skeletal muscle changes attributable to relatively increased β_2_-adrenoceptor activity [17]. These limitations, as well as species’ variations in Ca^2+^ cycling proteins and muscle fiber type composition, generally confound data interpretation and detract from these models. As noted earlier, some [18], [19], [21], [35], but not all studies in animal models [20], have uncovered definite, but variable, abnormalities in the ECC process. These intriguing findings in imperfect animal models mandate further investigations in skeletal muscle from HF patients.

### Physiological Ca^2+^ release in normal human skeletal fibers

To our knowledge there are no previous studies of the Ca^2+^ release evoked by AP stimulation in human skeletal muscle fibers in health or disease. Two reports of the Ca^2+^ release flux in healthy human fibers performed under voltage-clamp conditions have been published [36], [37]. Interestingly, though it would be expected that under these conditions maximal Ca^2+^ fluxes would be recorded, the values calculated in these reports were significantly smaller than ours, by a factor of at least 2-fold. Differences in the fibers’ integrity, the use of Ca^2+^ sensors, and calculation methodologies may partially explain these differences. In one study, percutaneous needle biopsies were obtained, yielding only short muscle segments [36]. It is possible that the short length of these fibers adversely affected their functional performance; in our experience electrophysiological integrity of the fibers increases with increasing biopsy length. In the other study [37], a very low rate of success per biopsy was reported, again raising the question of the fibers’ integrity. In general, the recording of normal electrical activity and larger Ca^2+^ release fluxes suggests that our fibers were possibly in better physiological condition throughout the experimentation.

### Reduced Ca^2+^ release in fibers from HF patients

The most important and novel finding in our study is that the ECC process, specifically AP-evoked Ca^2+^ release, is impaired in fibers from the VL muscle of HF patients compared to those of healthy volunteers. We have previously reported a reduced expression of sarcoplasmic reticulum Ca^2+^-ATPase (SERCA2a) and dihydropyridine receptor (DHPR) in homogenates of VL muscle from HF patients compared to healthy volunteers, whereas SERCA1a and ryanodine receptor (RyR1) levels were not different [15]. The simplest predictions from those biochemical data would be that SR Ca^2+^ content is reduced in fibers from HF patients compared to those from healthy volunteers, and/or that there are orphaned RyR1s. Either alteration could result in a significant reduction of Ca^2+^ fluxes potentially leading to reduced exercise performance and increased fatigability characteristic of HF patients. Diminished peak Ca^2+^ release was present in response to single APs; a finding that would be expected to result in smaller peak twitch and tetanic tension (i.e. muscle weakness and premature fatigability). Further investigations are necessary to identify the exact step(s) in the ECC process that lead to this marked impairment in AP-evoked Ca^2+^ release in HF patients. Comparisons of the physiological trigger for Ca^2+^ release (the AP which initiates the ECC process) in healthy volunteers and HF patients were performed and essentially eliminated this as a potential cause (see discussion below).

### Electrical activity

The amplitude and duration of the transverse tubular system depolarization are key factors in determining the features of Ca^2+^ release. Our measurements demonstrate that the Ca^2+^ release in fibers from HF patients are triggered by unaltered APs. The constancy and similarity of AP amplitude in fibers from both healthy volunteers and HF patients is remarkable; since the “resting” potential is kept close to −90 mV in both fiber groups, this result implies that the AP overshoot was also similar between (i.e. circa +54 mV).

The maximum depolarization rates of the AP in control and HF fibers are similar to that found in frog fibers using a similar method [24]; nevertheless, we found that the rate of depolarization is larger in HF fibers. There may be a number of physiological reasons for this difference, including differences in ionic channel endowment. It has been recently reported that the expression of the adult isoform of the Na^+^ channels (NaV1.4) is significantly increased in *soleus muscles* of rat models of muscle disuse, a condition to which HF patients may be prone [38], [39]. In fact, an important controversy in the HF literature is whether the skeletal myopathy in HF stems from simple disuse or from the HF condition characterized by neurohumoral activation and inflammation [39]. The increased expression of NaV1.4 might partially explain why paradoxically the weaker fibers of HF patients are electrically more rapid than those of healthy volunteers, lending support to the disuse position. Other possibilities, such as differences in specific capacitance, and/or fine geometry of the transverse tubular system (diameter and luminal resistance of the tubules) may also explain our findings, thus warranting further investigations on this topic.

### Ca^2+^ release results may be ubiquitous

Both the kinetics and magnitude of the Ca^2+^ transients have been reported to differ between fast and slow muscle fibers, and to correlate with the myosin heavy chain (MHC) type expressed; specifically, Ca^2+^ release rates are larger in type II than in type I fibers [40]. Exercise fatigue in HF patients has been suggested to result from fiber type transformation [10], [13], [41]; arguably, a decreased proportion of slow, aerobic, type I fibers with respect to the number of fast, anaerobic, fatigable, type IIx fibers in the locomotion muscles, such as the VL, might explain the fatigability phenotype. However, an increased proportion of type II fibers in which the ECC process is intact in HF patients would be expected to produce more powerful and quicker muscles; of course, this not the case. In our prior report in HF patients [15], we confirmed the previously reported fiber type shift [10], [13], [41]. We also reported the novel finding that SERCA2a and DHPR protein levels were decreased in homogenates of VL muscle in HF patients compared to healthy volunteers, whereas SERCA1a and ryanodine receptor (RyR1) levels were not different. Although the observed fiber shift could explain the decrease in SERCA2a, which is present only in type I fibers, it cannot explain either the decrease in DHPR level, since DHPR/RyR1 density is greater in type II than type I fibers in rodents [42], [43], or the constancy of the SERCA1a and RyR content.

Interestingly, we found a narrow, left-shifted distribution of peak Ca^2+^ fluxes in HF patients, without overlap with the peak Ca^2+^ fluxes in healthy volunteers, consistent with a ubiquitous abnormality affecting the ECC process in every fiber type (I and II) of VL muscles. In other words, the level of Ca^2+^ release from HF fibers with the greatest Ca^2+^ flux, (presumably type II fibers, also expected in larger proportions in the biopsies of HF patients), was even lower than the level of Ca^2+^ release from healthy fibers with the lowest Ca^2+^ flux, presumably type I fibers. A simple fiber-type shift (i.e. from type I to type II) should have resulted in an overall right-shifted distribution of HF fibers, not seen in Figure 4B; that is, a fiber shift would have been expected to produce a larger proportion of the HF fiber population displaying larger Ca^2+^ release.

In summary, our results strongly support the hypothesis that fibers from HF patients have marked and ubiquitous impairments in ECC, specifically AP-evoked Ca^2+^ release that affects all the fibers, independent of their type, in VL muscles.

### Limitations

The use of humans, in place of animals, for investigation, has its own limitations, which we acknowledge here: First, patients are on medications including statins, which, for ethical reasons, cannot be interrupted, and these medications may have systemic effects. Nonetheless, it is well established that despite optimal medical therapy HF patients have severe exercise limitations. We would argue that it is precisely these individuals who need to be studied, since despite the best medical therapy, their exercise capacity continues to be impaired. It is worth noting that no patient was diagnosed with a statin myopathy. Secondly, patients have diverse etiologies of HF, however, the severity of exercise dysfunction, and the characteristics of the skeletal myopathy, including the fiber type transformation, are known to be independent of the specific etiology of the HF [44],[45]. Finally, since the experiments in fibers from both HF patients and healthy volunteers were performed at room temperature, the absolute values of the Ca^2+^ release fluxes reported here are possibly smaller than those that could be obtained at normal body temperatures. Though future experimentation will take this into account, the observed impairments in Ca^2+^ release of fibers from HF patients are likely to represent a general limitation rather than an experimental peculiarity linked to the choice of temperature.

## Conclusion

We report for the first time in single fibers from HF patients compared to healthy volunteers, that AP-evoked Ca^2+^ release is significantly diminished in fibers obtained from the locomotive VL muscle in HF patients. This diminished Ca^2+^ release is not attributable to significant alterations in the AP of fibers from HF patients compared to fibers from healthy volunteers, since the electrical activity in both groups is virtually identical. Our previous findings of diminished DHPR and SERCA2 expression are suggestive of decreased SR Ca^2+^ content, and/or orphaned RyR1; the relative contributions of these abnormalities and others in the ECC process await further investigation. This decrease in Ca^2+^ flux was present in every fiber tested, consistent with a ubiquitous abnormality involving the ECC process in every fiber type in the VL muscle of HF patients, and unlikely to be attributable to a simple fiber type shift. Finally, using this approach, Ca^2+^ fluxes in individual fibers can be compared between muscles of locomotion, vulnerable to disuse, and compared to fibers in non-locomotive muscles, such as the deltoid, to assess if abnormalities of the ECC process are a systemic problem, attributable to systemic neurohumoral activation and inflammation, or a localized problem, more easily explained by disuse. The impact of exercise training on the impairment of ECC and Ca^2+^ release has widespread clinical relevance, both in understanding the role of disuse in this impairment, as well as an important therapeutic approach, and warrants further study.

## Supporting Information

Figure S1 **The transverse tubular system in live fibers from control and HF individuals.** Muscle bundles dissected from biopsies obtained from control individuals (A) and HF patients (B) were stretched to a sarcomere length similar to that in the electrophysiological experiments and pinned down on a Sylgard bottomed dish. The fibers were stained with di-8-ANEPPS and imaged using a two-photon laser scanning microscope (Biorad Radiance 2000). The dye was excited with a 920 nm laser beam, and the emission was collected using a dichroic/band-pass combination of 506 nm/620–650 nm. It can be seen in the optical section in A that, as previously described for other mammalian fibers, di-8-ANEPPS clearly demonstrate the regular double row pattern of the transverse tubular system. The surface membrane can also be seen at the right upper part of the image. The image in B demonstrate that the transverse tubular system of fibers from HF patients is indistinguishable from that of control fibers. The scale represent 15 µm in both images.
(EPS)
